# When the Mind Cannot Shift: Cognitive Flexibility Impairments in Methamphetamine-Dependent Individuals

**DOI:** 10.3390/bs15091207

**Published:** 2025-09-05

**Authors:** Xikun Zhang, Yue Li, Qikai Zhang, Yuan Wang, Jifan Zhou, Meng Zhang

**Affiliations:** Department of Psychology and Behavioral Sciences, Zhejiang University, Hangzhou 310058, China; xikun_zhang@zju.edu.cn (X.Z.); 22339059@zju.edu.cn (Y.L.); 12439013@zju.edu.cn (Q.Z.); 22439011@zju.edu.cn (Y.W.)

**Keywords:** cognitive flexibility, methamphetamine-dependent, task switching, WCST

## Abstract

Cognitive flexibility—the ability to adapt cognitive strategies and behavioral responses in changing environments—is a key component of executive function, supporting rule updating and conflict resolution. Individuals with substance addiction often exhibit behavioral rigidity and reduced adaptability, reflecting impairments in this domain. This study examined cognitive flexibility in individuals with methamphetamine dependence through three behavioral tasks—intra-dimensional task switching, extra-dimensional task switching, and the Wisconsin Card Sorting Test (WCST)—in combination with a subjective self-report measure. Results showed that, compared to healthy controls, methamphetamine-dependent individuals demonstrated elevated reaction time switch costs in Intra-dimensional Task Switching and increased accuracy switch costs in Extra-dimensional Task Switching, as well as more perseverative and non-perseverative errors in the WCST. These findings suggested not only reduced performances in explicitly cued rule updating and strategic shifting but also deficits in feedback-driven learning and inflexibility in cognitive set shifting on methamphetamine-dependent individuals. Moreover, their self-reported cognitive flexibility scores were aligned with their objective performance, significantly lower than healthy controls. In summary, these findings revealed consistent cognitive flexibility impairments at both behavioral and subjective levels in individuals with methamphetamine dependence, indicating a core executive dysfunction that may undermine adaptive functioning in real-life contexts. The study offers critical insights into the cognitive mechanisms underlying addiction and provides a theoretical foundation for targeted cognitive interventions.

## 1. Introduction

Cognitive flexibility refers to the capacity to adjust cognitive strategies and behavioral responses in accordance with changing environmental demands and task requirements (Braem & Egner, 2018; Dajani & Uddin, 2015; Ionescu, 2017). This capacity involves inhibiting ineffective responses, reallocating attentional resources, and constructing new mental representations (Dajani & Uddin, 2015; Miyake & Friedman, 2012). It is a core executive function that supports higher-order processes such as rule updating, and conflict resolution (Diamond, 2013), as well as attentional shifting, set maintenance, inhibition of prepotent responses, and flexible strategy use in complex and changing environments (Dajani & Uddin, 2015; Miyake & Friedman, 2012; Monsell, 2003; Uddin, 2021). Fundamentally, cognitive flexibility functions as an adaptive regulatory mechanism that enables individuals to adjust to contexts requiring behavioral inhibition and strategy switching—such as regulating impulsive responses to craving cues—through attentional shifting, strategy reconfiguration, and cognitive updating (Braem & Egner, 2018; Dajani & Uddin, 2015).

Extensive research has demonstrated that deficits in cognitive flexibility are prevalent across various psychiatric and neurodevelopmental disorders, including autism spectrum disorder (Andreou et al., 2022; Lacroix et al., 2024; Landry & Al-Taie, 2016), obsessive–compulsive disorder (Gruner & Pittenger, 2017; Kanen et al., 2019; Rosa-Alcázar et al., 2020; Vaghi et al., 2017; Whitton et al., 2014), schizophrenia (Everett et al., 2001; Thoma et al., 2007), major depressive disorder (Pan et al., 2019; Snyder, 2013), attention-deficit/hyperactivity disorder (Rommelse et al., 2007; Willcutt et al., 2005; Yeung et al., 2022), and substance use disorders (Barrado-Moreno et al., 2025; Izquierdo & Jentsch, 2012; Leeman & Potenza, 2012; Ramey & Regier, 2019), etc. Individuals with such conditions often exhibit cognitive rigidity and perseverative responding, struggling to modify behavioral strategies when task demands shift. As Diamond (2013) has emphasized, cognitive flexibility is a fundamental component of executive function, and its impairment can severely limit individuals’ capacity to cope with complex real-world scenarios, leading to inflexible, repetitive, and maladaptive behavior.

In the field of addiction research, cognitive dysfunction is increasingly recognized as a central mechanism underlying both the development and persistence of addictive behaviors (Ornstein, 2000). Specifically, impairments in the capacity to update strategies and adjust responses—core elements of cognitive flexibility—are considered key to understanding the compulsivity observed in addiction (Goldstein & Volkow, 2011; Moeller et al., 2024; Moeller & Goldstein, 2014; Woicik et al., 2009). Empirical findings showed that individuals with substance use disorders often demonstrate diminished performance on executive function tasks, including increased perseveration (Barceló & Knight, 2002; Bickel et al., 2012; Farhadian et al., 2017; Fernández-Serrano et al., 2010a; Hester & Garavan, 2004), impaired rule shifting, and reduced sensitivity to feedback (Fernández-Serrano et al., 2010a). These deficits may compromise their ability to adjust behavior appropriately even when they are aware of the harmful consequences of their actions, creating a cognitive paradox of “knowing but unable to stop.” 

In line, impairments in cognitive flexibility have been documented across users of various substances, including alcohol (Barrado-Moreno et al., 2025; Corbit & Janak, 2016; Fernández-Serrano et al., 2010a, 2010b), nicotine (Luijten et al., 2020; Meier et al., 2024), cocaine (Colzato et al., 2009; Ersche et al., 2016; Fernández-Serrano et al., 2010a; Hester & Garavan, 2004; Kübler et al., 2005), and opioids (Gharahi et al., 2023). For instance, studies employing the Wisconsin Card Sorting Test (WCST, Barceló & Knight, 2002; Faustino et al., 2021; Feldstein et al., 1999) and similar paradigms consistently found that substance users exhibit higher rates of perseverative errors, reduced rule-shifting efficiency, and poor utilization of feedback (Fernández-Serrano et al., 2010b). In particular, methamphetamine has emerged as one of the most widely abused synthetic stimulants, with growing evidence of its damaging effects on executive functions (Basterfield et al., 2019; Farhadian et al., 2017; Sabrini et al., 2019).

Methamphetamine-dependent individuals have shown pronounced deficits in cognitive functions across multiple paradigms, including the Wisconsin Card Sorting Test (WCST), Stroop task, and n-back tasks—manifesting slower response times, increased error rates, and difficulties in updating cognitive strategies (Farhadian et al., 2017; Kofman et al., 2006; Sabrini et al., 2019). Neuroimaging studies further revealed significant reductions in dopamine D2 receptor availability among methamphetamine-dependent individuals, which strongly correlates with their severity of cognitive impairments (Dean et al., 2013; Jiang et al., 2024; Sabrini et al., 2019). Notably, these cognitive deficits may persist during prolonged abstinence, suggesting long-lasting neurocognitive dysfunction (Koob & Volkow, 2016; Stavro et al., 2013; Verdejo-García et al., 2019).

Despite accumulated evidence, recent systematic reviews and meta-analyses have highlighted that impairments in cognitive flexibility are a consistent and transdiagnostic feature of substance use disorders, yet the methodological approaches employed remain heterogeneous and often limited in scope (Smith et al., 2014; Wu et al., 2024). In particular, these syntheses emphasize the need for multi-method assessments that can disentangle different dimensions of flexibility, rather than relying solely on single-task paradigms. Building on this literature, two major gaps remain in the current field. First, most existing studies rely on a single, general-purpose task (e.g., only WCST), which fails to capture the multifaceted nature of cognitive flexibility—including both explicit rule-guided shifting and implicit learning from feedback. Secondly, few studies integrate both subjective self-reports and objective behavioral measures, leaving the subjective experience of cognitive inflexibility among methamphetamine-dependent individuals underexplored. These limitations hinder a comprehensive understanding of executive dysfunction in methamphetamine-dependent individuals and constrain the development of personalized cognitive interventions.

To address these issues, the present study developed a multi-indicator, dual-level assessment research to examine cognitive flexibility among individuals with methamphetamine dependence. Via three cognitive tasks and a self-report measure, we evaluated both objective and subjective dimensions of cognitive flexibility across different cognitive demands in methamphetamine-dependent individuals. Task 1 and 2 employed explicit rule-switching paradigms—intra-dimensional and extra-dimensional task switching (Kiesel et al., 2010; Monsell, 2003)—which required participants to adjust to clearly cued rules. These paradigms primarily assessed top-down executive functions such as deliberate strategy shifting, rule updating, and conflict resolution. We expected individuals with methamphetamine dependence to exhibit increased switch costs, reflecting impairments in guided cognitive adjustment even under structured conditions. Task 3 used the WCST, which emphasized implicit, feedback-driven adaptation. Unlike Task 1 and 2, the WCST required participants to infer shifting rules from feedback without explicit instruction, thereby targeting flexible set-shifting under uncertainty (Faustino et al., 2021; Feldstein et al., 1999). We anticipated elevated perseverative errors in the methamphetamine group, indicative of poor feedback utilization and increased cognitive rigidity. In parallel, the Chinese version of the Cognitive Flexibility Inventory (CFI; Wang et al., 2016) was used to assess participants’ self-perceived cognitive adaptability in real-world contexts. By integrating subjective and performance-based indicators, this study aimed to generate a comprehensive profile of flexibility-related dysfunction and inform the development of targeted cognitive interventions for addiction populations.

## 2. Materials and Methods

### 2.1. Participants

Methamphetamine-Dependent Group: A total of 47 male participants in the methamphetamine-dependent group were recruited from a compulsory drug rehabilitation center in Zhejiang Province, China. All participants met the diagnostic criteria for methamphetamine use disorder as defined in the DSM-V (American Psychiatric Association, DSM-5 Task Force, 2013). They all had a documented history of methamphetamine use and were undergoing withdrawal following the completion of physical detoxification.

Control Group: The control group consisted of 41 male participants who were recruited through monetary incentives and had no history of methamphetamine use, other substance use, or behavioral addiction.

All participants met the following inclusion criteria: normal or corrected-to-normal vision; normal color vision; right-handedness; aged between 18 and 45 years; no recent major family crises; and no history of psychiatric disorders. As the rehabilitation center only admits men, both the Methamphetamine-Dependent group and its Control group consisted exclusively of male participants, ensuring gender consistency in the study. Demographic characteristics of all participants are presented in Table 1.

### 2.2. Apparatus

All the experimental tasks were programmed by PsychoPy (version 2022.2.0) and administered on a desktop computer with a 24-inch LCD monitor (screen resolution: 1920 × 1080 pixels). Participants were seated at a viewing distance of approximately 60 cm, resulting in a visual angle of approximately 47.78° × 27.98°. All responses were recorded via a standard keyboard, and participants completed the task individually under controlled laboratory conditions.

### 2.3. Measurements and Procedure

To provide a multidimensional assessment of cognitive flexibility, this study employed a combined methodological approach incorporating both objective and subjective measures. Upon arrival at the laboratory, participants received a detailed explanation of the study and provided written informed consent. Those in the methamphetamine-dependent group should complete a Drug Use History Questionnaire to verify substance use details.

**Figure 1:**
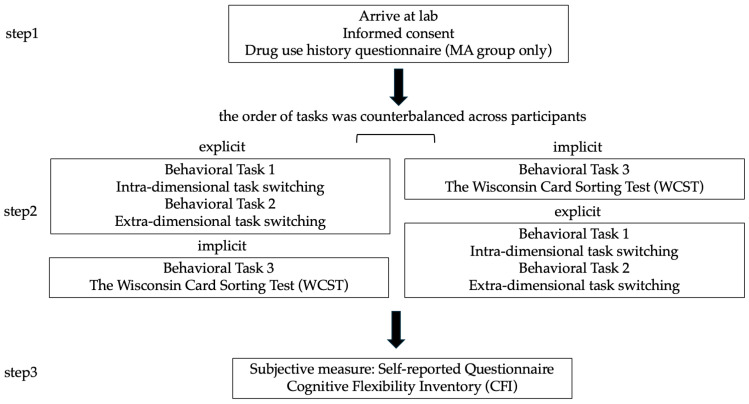
Experimental Procedure.

All subsequent procedures were conducted in a quiet testing environment under the supervision of trained experimenters. Specifically, participants first completed three behavioral tasks: (1) an intra-dimensional task-switching paradigm evaluating basic flexibility under low-complexity, explicitly cued conditions; (2) an extra-dimensional switching paradigm probing cross-domain rule shifting under higher cognitive load; and (3) the Wisconsin Card Sorting Test (WCST), assessing implicit, feedback-guided flexibility in unstructured contexts. To control for potential learning or fatigue effects, the order of the tasks was counterbalanced across participants. Following these tasks, the Cognitive Flexibility Inventory (CFI, Dennis & Vander Wal, 2010) was administered to capture participants’ subjective, metacognitive perceptions of flexibility in daily life. A schematic overview of the experimental procedure is presented in Figure 1.

#### 2.3.1. Behavioral Task 1: Intra-Dimensional Task Switching

Task 1 employed a single-factor between-subjects design, with group (methamphetamine-dependent vs. healthy control) as the independent variable. The dependent variables were intra-dimensional task-switching indices, that is, reaction time switch cost and accuracy switch cost.

The experimenter confirmed participants’ comprehension through verbal feedback before initiating the practice phase. Participants completed eight practice trials (excluded from the final analysis) and were required to achieve an accuracy rate of at least 75% to continue. Only those demonstrating adequate task understanding and response proficiency proceeded to the formal task. To ensure procedural consistency and data integrity, all sessions were conducted by the same experimenter.

**Figure 2:**
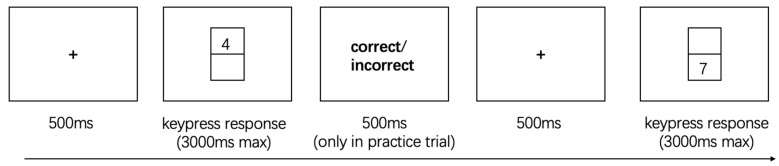
The Intra-Dimensional Task Switching.

The intra-dimensional task-switching paradigm is illustrated in Figure 2. The task involved classifying digits 1, 2, 3, 4, 6, 7, 8, and 9, with digit 5 excluded to avoid ambiguity in magnitude judgments. On each trial, a single digit appeared either above or below a central fixation square on the screen. When the digit appeared above the square, participants performed a parity judgment: pressing “M” for even numbers and “Z” for odd numbers. When the digit appeared below the square, participants performed a magnitude judgment: pressing “M” for numbers less than 5 and “Z” for numbers greater than 5. During the practice trials, participants received immediate feedback on the accuracy of their responses to ensure task understanding. In the formal experimental trials, no feedback was provided, and participants relied solely on explicit rule cues indicated by the digit’s position on the screen.

The task included two trial types: Repeat trials, in which the current task was identical to the previous trial (i.e., the digit appeared in the same spatial location and required the same judgment rule: parity or magnitude). Switch trials, in which the task differed from the preceding trial (i.e., the digit changed position, requiring a rule switch between parity and magnitude judgments) (Monsell, 2003).

The formal task consisted of two blocks, each comprising 64 trials. Each trial had a maximum response window of 3000 ms. The first trial of each block was excluded from statistical analyses, as it lacked a preceding context and therefore could not be classified as either a switch or repeat trial. The task was designed to ensure an equal number of parity and magnitude judgment trials, with a balanced proportion of switch and repeat trials distributed across the two blocks. Within each block, trial sequences were presented in a pseudo-randomized order, minimizing predictability and reducing potential practice or expectancy effects.

This intra-dimensional switching paradigm provides a basic behavioral index of cognitive flexibility, allowing for the assessment of individuals’ efficiency in rule updating and cognitive set-shifting under low-complexity conditions with explicit cues.

#### 2.3.2. Behavioral Task 2: Extra-Dimensional Task Switching

Task 2 utilized a similar single-factor between-subjects design to Task 1, with group (methamphetamine-dependent vs. healthy control) as the independent variable. The dependent measures were reaction time and accuracy switch costs derived from an extra-dimensional task-switching paradigm.

**Figure 3:**
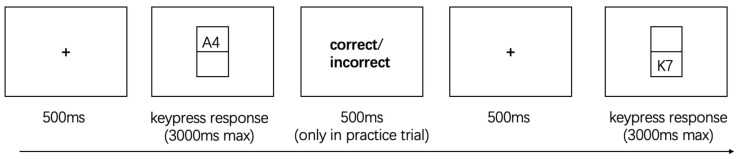
The Extra-Dimensional Task Switching.

As shown in Figure 3, stimuli consisted of letter-digit pairs (e.g., “A7,” “K2”), with letters drawn from a fixed set of vowels (A, E, O, U) and consonants (excluding “I” to avoid confusion with the digit “1”), and digits from 1, 2, 3, 4, 6, 7, 8, and 9. On each trial, the pair was displayed either in the upper or lower region of the screen, determining the task type.

On each trial, a letter-digit pair was presented either in the upper or lower rectangular region of the screen. When the pair appeared in the upper region, participants were instructed to classify the letter as either a vowel or a consonant, pressing the “M” key for vowels and the “Z” key for consonants. When the pair appeared in the lower region, participants classified the digit as either odd or even, with “M” corresponding to odd numbers and “Z” to even numbers. Consistent with Task 1, practice trials provided immediate feedback to ensure participants understood the task rules, while no feedback was given during the formal experimental trials, requiring participants to rely solely on explicit positional cues to guide their responses.

Trials were also categorized as repeated or switched based on the continuity of task type and screen location. Repeat trials involved consecutive judgments of the same type (e.g., two letter classifications), whereas switch trials required participants to shift from one task type to the other (e.g., from letter to digit judgment or vice versa), with the stimulus appearing in a different screen region (Monsell, 2003).

Each participant completed two blocks of 64 trials, with a 3000 ms response window per trial. Pseudo-randomized trial sequences ensured balanced distributions of switch/repeat types and minimized anticipatory effects. The first trial of each block was excluded from analysis due to the absence of a preceding context.

In contrast to the intra-dimensional switching task (Task 1), this paradigm required participants to alternate between two distinct cognitive domains—alphabetic and numeric—thereby placing greater demands on executive control. Specifically, it engaged higher-order processes such as task-set reconfiguration, cross-domain rule activation, and the inhibition of previously relevant strategies. Consequently, this task served as a more sensitive probe of advanced cognitive flexibility.

#### 2.3.3. Behavioral Task 3: Wisconsin Card Sorting Test (WCST)

The WCST, using a computerized version (WCST-128), was conducted as task 3. As in previous tasks, a single-factor between-subjects design was applied in task 3, with group (methamphetamine-dependent vs. healthy control) as the independent variable and standard WCST performance indices as dependent variables.

**Figure 4:**
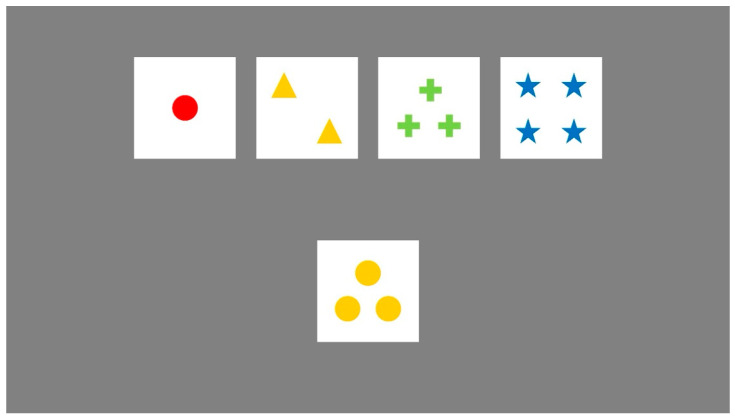
The Wisconsin Card Sorting Test.

As shown in Figure 4, participants were required to match a target card—randomly drawn from a deck of 128 unique cards—with one of four fixed reference cards. Each card varied along three dimensions: color (e.g., red, green, yellow, blue), shape (e.g., circle, triangle, star, cross), and number of items (1–4). However, participants were not informed which dimension governed the current sorting rule. After each selection, participants received feedback indicating whether the match was correct or incorrect. Based on this trial-by-trial feedback, they were expected to infer the underlying sorting rule and adjust their strategy accordingly. Once a participant achieved 10 consecutive correct responses, the sorting rule changed without warning, requiring them to detect the shift and flexibly adapt to the new rule. This process continued until all six rule shifts were completed or the entire deck was exhausted (Faustino et al., 2021; Feldstein et al., 1999).

Key behavioral indicators were recorded throughout the task to quantify cognitive flexibility. Perseverative errors captured failures to abandon an outdated rule despite negative feedback and served as a primary index of cognitive rigidity. Non-perseverative errors reflected incorrect responses unrelated to the previous rule, often indicating attentional fluctuations or unstable strategy use. Failures to maintain set referred to difficulties in consistently applying the correct rule before reaching the criterion for a rule shift, highlighting deficits in cognitive stability. The number of trials required to correctly identify the first sorting rule was also analyzed, providing insight into participants’ initial rule-learning and exploratory adaptability (Faustino et al., 2021).

Together, these indices enabled a more nuanced examination of participants’ ability to flexibly extract, maintain, and revise rules in response to ambiguous feedback. By comparing performances between the methamphetamine-dependent and control groups, the study extended the findings from the explicitly cued task-switching paradigms in Tasks 1 and 2 to a more ecologically valid, feedback-driven context of cognitive regulation.

#### 2.3.4. Subjective Cognitive Flexibility: Self-Reported Questionnaire

To evaluate perceived cognitive flexibility in daily life, participants completed the Chinese version of the Cognitive Flexibility Inventory (CFI), adapted from Dennis and Vander Wal (2010) by Wang et al. (2016). The CFI comprises 20 items rated on a 5-point Likert scale (1 = never, 5 = always), assessing individuals’ ability to reframe thoughts, adapt to challenges, and regulate behavior in everyday contexts. Items 2, 4, 7, 9, and 11 are reverse-scored.

Participants completed the paper-based questionnaire on site, with instructions to respond based on their recent real-life experiences. The CFI demonstrated excellent internal consistency across both groups (Cronbach’s α = 0.89), supporting its reliability in assessing subjective cognitive flexibility.

## 3. Results

### 3.1. Behavioral Task 1 Results: Intra-Dimensional Task Switching

To evaluate group differences in basic level of cognitive flexibility during intra-dimensional task switching, switch costs were calculated as follows: reaction time (RT) switch cost = RT_switch_ − RT_repeat_; accuracy switch cost = Accuracy_repeat_ − Accuracy_switch_. To minimize the influence of extreme values on group comparisons—particularly their potential to distort means and variances in a non-systematic manner—all Task 1 performance indicators were screened using a ±3 standard deviation (*SD*) criterion, following standard data-cleaning procedures in behavioral research (Tabachnick & Fidell, 2019). Data points exceeding this threshold were excluded from subsequent analyses. Given the significant age difference between the two groups (*p* < 0.001), age was included as a covariate in subsequent analyses, and ANCOVAs were conducted to compare the methamphetamine-dependent group and the control group on these two indices. Descriptive statistics and adjusted between-group comparisons are presented in Table 2.

After controlling for age in an ANCOVA, the methamphetamine-dependent group showed a marginally higher reaction time (RT) switch cost (*M* = 0.619, *SD* = 0.400) compared to the control group (*M* = 0.295, *SD* = 0.124), *p* = 0.09, partial *η*^2^ = 0.033, 95% CI [0.000, 0.137]. For accuracy switch cost, no significant group difference was found after adjusting for age (*M* = 0.035, *SD* = 0.086 for the methamphetamine-dependent group vs. M = 0.029, *SD* = 0.030 for the control group), *p* = 0.29, partial *η*^2^ = 0.013, 95% CI [0.000, 0.098].

**Figure 5:**
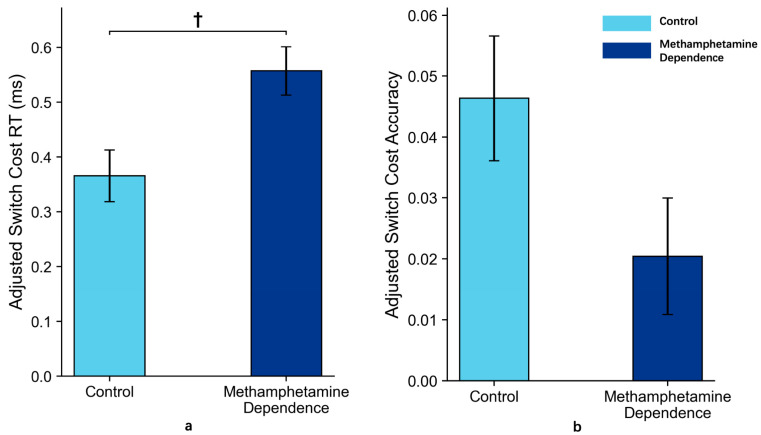
Switch Costs in Methamphetamine Dependence vs. Control Group in Task 1 (*M* ± *SE*). *Note*: Panel (**a**) shows the reaction time switch costs between the two groups after ANCOVA adjustment for age; Panel (**b**) shows the accuracy switch costs between the two groups after ANCOVA adjustment for age. Error bars represent standard errors. ‘†’ indicates marginal significance.

These results suggest that, after adjusting for age, individuals in the methamphetamine-dependent group exhibit a trend toward slower task switching under low-complexity, explicitly cued conditions, as reflected in RT switch costs. No such trend was observed for accuracy switch costs (Figure 5).

### 3.2. Behavioral Task 2 Results: Extra-Dimensional Task Switching

To evaluate group differences in advanced cognitive flexibility during extra-dimensional task switching, switch costs were calculated as follows: reaction time (RT) switch cost = RT_switch_ − RT_repeat_; accuracy switch cost = Accuracy_repeat_ − Accuracy_switch_. As in Task 1, all Task 2 data were screened using the same ±3 SD criterion to ensure comparability and robustness, and age was included as a covariate in the analyses. Descriptive statistics for extra-dimensional task switching in the methamphetamine-dependent group and the control group are presented in Table 3.

After controlling for age in an ANCOVA, the methamphetamine-dependent group showed a marginally higher accuracy switch cost (*M* = 0.024, *SD* = 0.030) compared to the control group (*M* = 0.009, *SD* = 0.037), *p* = 0.08, partial *η*^2^ = 0.035, 95% CI [0.000, 0.142]. For reaction time (RT) switch cost, no significant group difference was found after adjusting for age (*M* = 0.343, *SD* = 0.182 for the methamphetamine-dependent group vs. *M* = 0.369, *SD* = 0.155 for the control group), *p* = 0.43, partial *η*^2^ = 0.008, 95% CI [0.000, 0.084].

**Figure 6:**
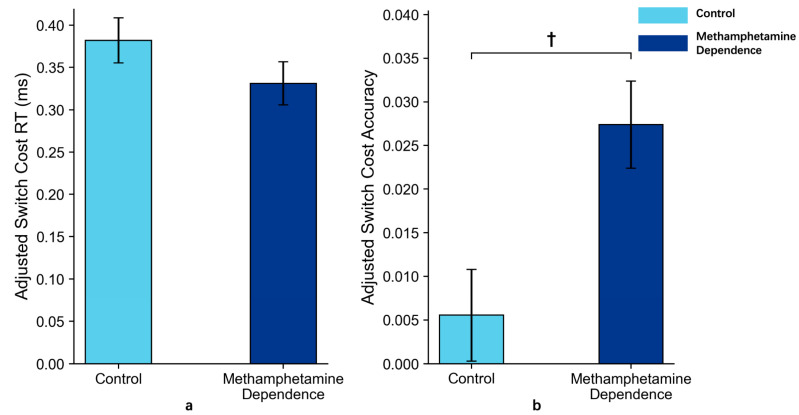
Switch Costs in Methamphetamine Dependence vs. Control Group in Task 2 (*M* ± *SE*). *Note*: Panel (**a**) shows the reaction time switch costs between the two groups after ANCOVA adjustment for age; Panel (**b**) shows the accuracy switch costs between the two groups after ANCOVA adjustment for age. Error bars represent standard errors. ‘†’ indicates marginal significance.

These results suggested that individuals with methamphetamine dependence may exhibit impaired advanced cognitive flexibility, as indicated by a marginally higher accuracy switch cost under explicitly cued, high-complexity conditions (*p* = 0.08). This trend reflects a potentially structured impairment in flexible cognitive control when shifting across cognitive domains (Figure 6).

### 3.3. Behavioral Task 3 Results: Wisconsin Card Sorting Test

In the WCST task, due to the emphasis on participants’ autonomous strategy exploration and behavioral performance across multiple rule shifts, individual differences in strategy use may lead to extreme deviations in certain dimensions (e.g., very high error counts or zero completed categories). Preliminary data screening revealed that some participants showed substantial deviations from the sample mean on several indicators, reflecting a pronounced long-tail distribution. As in Task 1, all WCST performance indicators were also screened using the same ±3 SD criterion as applied in Tasks 1 and 2, ensuring uniform data-cleaning procedures across tasks (Tabachnick & Fidell, 2019), and ANCOVAs were conducted on core WCST indicators with age included as a covariate to compare the methamphetamine-dependent group and the healthy control group.

The descriptive statistics of the Wisconsin Card Sorting Test (WCST) performance for the methamphetamine-dependent group and the control group are presented in Table 4.

After controlling for age in an ANCOVA, for perseverative errors, the methamphetamine-dependent group showed a marginally higher error rate *(M* = 2.738, *SD* = 1.499) compared to the control group (*M* = 1.659, *SD* = 1.237), *p* = 0.08, partial *η*^2^ = 0.036, 95% CI [0.000, 0.146]. This marginal trend suggests potential difficulty in rule updating and a tendency to persist in applying outdated strategies—particularly in the absence of explicit cues. For non-perseverative errors, after adjusting for age, the methamphetamine-dependent group exhibited significantly higher error rates (*M* = 10.262, *SD* = 3.513) compared to the control group (*M* = 8.100, *SD* = 3.087), *p* = 0.004, partial *η*^2^ = 0.099, 95% CI [0.011, 0.236]. This finding indicates attentional instability or inconsistent strategy use under ambiguous conditions, with a medium effect size suggesting meaningful group differences in non-perseverative cognitive control. For trials to complete the first category, no significant group difference was found between the methamphetamine-dependent group (*M* = 6.310, *SD* = 0.897) and the control group (*M* = 6.122, *SD* = 0.900), *p* = 0.94, partial *η*^2^ = 6.611 × 10^−5^, 95% CI [0.000, 1.361 × 10^−4^], suggesting that both groups demonstrated relatively comparable concept formation abilities during the initial learning phase. Similarly, for failures to maintain set, the two groups did not differ significantly (*M* = 0.366 vs. 0.488), *p* = 0.123, partial *η*^2^ = 0.030, 95% CI [0.000, 0.136], indicating comparable performance in maintaining task demands.

### 3.4. Subjective Results: Self-Reported Questionnaire

**Figure 7:**
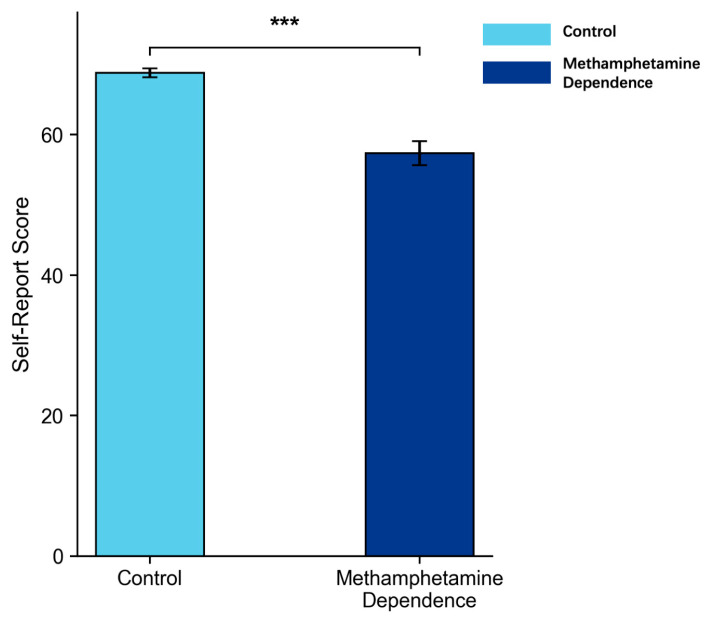
Cognitive Flexibility Inventory Self-Report Scores in Methamphetamine Dependence vs. Control Group (*M* ± *SE*). *Note*: Error bars represent standard errors. *** *p* < 0.001.

In the analysis of subjective cognitive flexibility, we first screened the CFI data for response validity. Consistent with established criteria for detecting invalid self-report data (Meade & Craig, 2012), five participants from the methamphetamine-dependent group were excluded because their responses showed extreme patterns—specifically, uniformly endorsing the same response option (e.g., “never” or “always”) across all 20 items, resulting in zero variance in item-level responses. Such patterns are widely considered to indicate inattentive or non-differentiated responding and therefore compromise the interpretability of scale scores. ANCOVA was then conducted on the remaining participants with age as a covariate, revealing that the methamphetamine-dependent group reported significantly lower cognitive flexibility scores than the control group (*M* = 57.34 vs. 72.45), *p* < 0.001, partial *η*^2^ = 0.29, 95% CI [0.140, 0.431], as illustrated in Figure 7.

These findings revealed a pronounced reduction in self-perceived cognitive flexibility among individuals with a history of methamphetamine use. Rather than directly mirroring objective task performance, this self-reported impairment likely reflects metacognitive awareness—namely, a subjective recognition of difficulties in regulating thought and behavior in everyday contexts.

## 4. Discussion

Cognitive flexibility—the core capacity that enables individuals to adapt to changing environments by adjusting thinking patterns and behavioral strategies—plays a critical role in the development and maintenance of addictive behaviors (Braem & Egner, 2018; Cañas et al., 2003; Dajani & Uddin, 2015; Koch et al., 2018; Uddin, 2021). This study systematically evaluated cognitive flexibility in individuals with methamphetamine dependence through a multi-method approach, including three objective cognitive tasks: intra-dimension task switching, extra-dimension task switching (Monsell, 2003), and the Wisconsin Card Sorting Test (WCST, Faustino et al., 2021) and a subjective self-report questionnaire (Dennis & Vander Wal, 2010). Overall, the findings revealed objective impairments in cognitive flexibility in the methamphetamine-dependent group, while self-reported difficulties indicated a metacognitive awareness of these challenges. This convergence between behavioral deficits and perceived difficulties underscores the ecological and clinical relevance of the results (Cañas et al., 2003, see Table 5).

Behavioral evidence of cognitive inflexibility emerged consistently across three tasks that varied in cognitive complexity, rule structure, and feedback requirements, underscoring the robustness of this dysfunction. Although many of the individual effects reached only marginal significance, taken together, these converging findings suggest a reliable pattern of impairment. Importantly, each task targeted a distinct yet complementary dimension of cognitive flexibility, allowing for a more nuanced understanding of how these impairments manifest.

Tasks 1 and 2 both assessed cognitive flexibility under explicitly cued conditions using task switching paradigm, providing a structured evaluation of participants’ ability to update and shift cognitive sets when task demands changed (Dreisbach & Mendl, 2024; Kiesel et al., 2010). Task 1, employing intra-dimensional task switching (e.g., shifting from parity to magnitude judgments of digits), reflected basic cognitive flexibility under low-complexity, within-domain conditions (Monsell, 2003). Results showed that individuals with methamphetamine dependence exhibited marginally elevated reaction time switch costs, suggesting slowed rule updating even when demands were minimal.

Task 2 increased complexity by requiring extra-dimensional task switching across cognitive domains (e.g., shifting between letter and digit classifications). Here, the methamphetamine-dependent group demonstrated marginally greater accuracy switch costs, indicating difficulty in rule selection and implementation when cross-domain shifting was required. This pattern suggests a more structured, capacity-level deficit in cognitive flexibility, likely reflecting limitations in the ability to maintain rules, exert inhibitory control, and monitor performances (Egner & Siqi-Liu, 2024; Richter & Yeung, 2015).

Together, these findings indicate that individuals with methamphetamine dependence exhibit broad impairments in cognitive flexibility under structured conditions, with Task 1 highlighting slowed processing during basic set-shifting and Task 2 revealing failures in accurate rule application under higher task complexity. These results provide consistent evidence that cognitive inflexibility in this population is evident across varying demands, underscoring its relevance as a target for intervention (Cañas et al., 2003; Schwarze et al., 2024).

In contrast, Task 3 employed the Wisconsin Card Sorting Test (WCST) to assess cognitive flexibility from the perspective of feedback-driven, implicit strategy updating. Unlike explicit rule-based tasks (task 1 and 2), the WCST requires participants to independently explore, learn, and continuously adjust their categorization strategies in the absence of clearly defined rules. The task emphasizes the integrated regulation of feedback utilization, rule maintenance, and set shifting (Barceló & Knight, 2002; Feldstein et al., 1999). Results showed that methamphetamine-dependent participants made marginally more perseverative errors—defined as the tendency to persist in applying a previously valid but currently incorrect sorting rule despite receiving negative feedback (Faustino et al., 2021)—suggesting a notable deficit in adaptive feedback integration. This pattern aligns with the updating difficulties observed in the task-switching paradigms, providing converging evidence of impaired cognitive flexibility. At the same time, no significant group differences were observed in Failures to Maintain Set or Trials to Complete First Category. This suggests that, once methamphetamine-dependent individuals successfully identified the correct sorting rule, their ability to maintain it was relatively preserved, consistent with prior findings that maintenance processes are less disrupted than set-shifting in addiction populations (Verdejo-García et al., 2004). Similarly, the comparable performance on Trials to Complete First Category indicates that both groups were able to grasp the task demands and infer the initial sorting principle, likely aided by the explicit task instructions. Taken together, these results suggest that the primary deficit in methamphetamine dependence lies not in initial rule acquisition or rule maintenance but rather in flexibly updating and shifting cognitive sets in response to changing feedback, as reflected in perseverative errors and task-switching costs. Notably, when comparing effect sizes across tasks, the WCST yielded a slightly larger impairment relative to the task-switching paradigms, suggesting that feedback-driven rule updating may serve as a more sensitive indicator of cognitive inflexibility in methamphetamine dependence. Moreover, these findings suggest that cognitive rigidity in this population extends beyond explicit rule-guided contexts to feedback-driven situations requiring flexible adjustment under uncertainty, which may hinder disengagement from maladaptive behaviors and increase relapse risk when encountering addiction-related cues (Brecht & Herbeck, 2014; Courtney & Ray, 2014; Farhadian et al., 2017; Sabrini et al., 2019).

The behavioral task findings of the present study provide clear evidence of impaired cognitive flexibility in individuals with methamphetamine dependence. These impairments are further supported by compelling neurobiological evidence. At the neural level, cognitive flexibility deficits in addiction have been consistently linked to dysfunction in prefrontal–striatal circuits and dysregulation of dopamine transmission (Goldstein & Volkow, 2011). Neuroimaging studies have demonstrated reduced gray matter volume, disrupted white matter integrity, and abnormal functional connectivity in prefrontal regions among individuals with substance use disorders—areas critical for flexible cognitive control (Uddin, 2021; Woicik et al., 2009; Zilverstand et al., 2018). Importantly, recent neuroimaging research in methamphetamine-dependent individuals specifically has shown prefrontal cortical thinning (Thompson et al., 2004), decreased frontostriatal connectivity linked to executive dysfunction (Salo et al., 2009), and abnormal error-related activity in the anterior cingulate cortex during cognitive control tasks (London et al., 2015). More recent work has further revealed disrupted salience network connectivity associated with craving and impulsivity in abstinent methamphetamine users (Lai et al., 2024), highlighting alterations in large-scale brain networks that underlie adaptive control and flexible behavior. Additionally, imbalances in neurotransmitter systems, particularly those involving dopamine, glutamate, and GABA, have been implicated in impaired behavioral regulation and executive dysfunction within addiction populations (Botvinick & Braver, 2015). Together, these behavioral and neurobiological findings underscore the presence of robust and multifaceted impairments in cognitive flexibility among individuals with methamphetamine dependence. However, beyond these objective deficits, it is equally important to consider how methamphetamine-dependent individuals perceive and evaluate their own cognitive adaptability in daily life, as this metacognitive perspective can provide additional insights into both the lived experience of cognitive challenges and potential avenues for intervention.

From a subjective perspective, individuals with methamphetamine dependence reported significantly lower cognitive flexibility on the Cognitive Flexibility Inventory (CFI; Dennis & Vander Wal, 2010) compared to healthy controls. While there is a trend toward impaired objective capacity for flexible cognitive control, their metacognitive awareness of these difficulties appears relatively intact, potentially due to the tangible impact of cognitive challenges on daily life and the structured feedback received in rehabilitation settings (Basterfield et al., 2019). This preserved insight suggests that methamphetamine-dependent individuals recognize their cognitive challenges and may be receptive to interventions aimed at enhancing flexibility and adaptive functioning. Clinically, this implies that cognitive flexibility training—whether through computerized cognitive remediation, strategy-based interventions, or metacognitive approaches—may be a promising adjunct to standard rehabilitation programs. By strengthening the capacity to shift perspectives and disengage from rigid routines, such interventions could support adaptive behavior change and reduce relapse risk in methamphetamine-dependent populations.

Taken together, the present findings provide a comprehensive profile of cognitive flexibility in methamphetamine dependence. They demonstrate deficits spanning multiple levels of complexity, from basic, explicitly cued rule switching to feedback-guided, implicit strategy updating, indicating a broad dysfunction in flexible cognitive control with full awareness of these difficulties. These insights emphasize not only the theoretical significance of cognitive inflexibility in addiction but also its practical implications, highlighting cognitive flexibility as a key treatment target to promote recovery.

Despite its strengths, this study has several limitations. First, the demographic composition of the sample constrains the generalizability of the findings. Due to logistical and access constraints, only male participants were recruited, and the methamphetamine-dependent group was on average substantially older than the control group. Both factors may have influenced the observed outcomes. Gender has been shown to modulate cognitive flexibility, suggesting that excluding female participants may overlook meaningful sex differences (García-Fernández et al., 2025; Gargiulo et al., 2022; LaClair et al., 2019). Likewise, age-related declines in processing speed and executive functioning are well documented and may have contributed to group differences independent of substance use. Although we statistically controlled for age in all behavioral analyses, residual confounding cannot be entirely excluded. Moreover, as participants were recruited exclusively from rehabilitation centers, the findings may not fully generalize to other methamphetamine users, such as those not currently engaged in treatment or at different stages of substance use. Second, task order effects were observed, as participants’ performance improved from Task 1 to Task 2. Although we counterbalanced the order of the task-switching paradigms with the WCST, all participants completed Task 1 before Task 2. This likely introduced practice-related gains. Future studies should employ more rigorous counterbalancing procedures or incorporate longer intervals between tasks to further minimize such effects. Third, lifestyle-related confounds such as smoking—which is highly prevalent among substance-using populations and has been linked to executive dysfunction—were not systematically assessed. The omission of such factors may have influenced the observed findings and should be carefully addressed in future research. Fourth, the present study relied exclusively on behavioral paradigms to assess cognitive flexibility. Although these tasks provide valuable insights into executive functioning, they do not directly capture the neural mechanisms underlying the observed deficits. Future research would benefit from integrating neuroimaging or electrophysiological techniques (e.g., fMRI, EEG) to explore the brain circuits associated with flexible cognitive control in addiction populations. Furthermore, the cross-sectional design of the present study precludes causal inference. It remains unclear whether the observed cognitive inflexibility reflects a pre-existing vulnerability that predisposes individuals to methamphetamine use or a neurocognitive consequence of chronic drug exposure. Longitudinal and prospective designs will be essential to disentangle these possibilities.

Finally, while task-switching paradigms and the WCST allow for rigorous experimental control, their ecological validity remains limited when extrapolating to real-world manifestations of cognitive flexibility. Flexible behavior in daily life often involves more complex, socially embedded, and emotionally salient contexts than those captured by laboratory tasks. Moreover, different types of cognitive flexibility paradigms may tap into distinct underlying processes. For instance, recent research on multimodal task switching—requiring shifts across sensory modalities such as visual–auditory or visuo-motor—suggests that such paradigms engage additional mechanisms, including cross-modal integration and modality-specific attentional control, that may not be fully captured by unimodal switching tasks (Kiesel et al., 2010; Koch et al., 2018; Wasylyshyn et al., 2011). Thus, while the present study employed intra-dimensional, extra-dimensional, and feedback-driven rule-shifting paradigms to cover complementary aspects of cognitive flexibility, our findings should be interpreted within the scope of these task choices. Future research integrating multimodal, social, or affective switching paradigms will be essential to establish the generalizability and ecological validity of cognitive flexibility impairments in methamphetamine dependence.

## 5. Conclusions

This study systematically evaluated cognitive flexibility in individuals with methamphetamine dependence using a multi-method approach. Behaviorally, individuals with methamphetamine dependence demonstrated a trend toward increased reaction time switch costs during intra-dimensional task switching, marginally higher accuracy switch costs during extra-dimensional task switching, and elevated perseverative and non-perseverative errors in the WCST. Consistently, they reported lower perceived cognitive flexibility, indicating awareness of these difficulties in daily life. Together, these converging findings point to a pattern of cognitive inflexibility in methamphetamine-dependent individuals, with implications for interventions targeting flexible cognitive control to support behavioral change.

## Figures and Tables

**Table 1:** Demographic information and clinical characteristics of participants.

	Methamphetamine-Dependent Group ^a^	Control Group ^a^	*t*
Sample Size	47	41	/
Gender	all males	all males	/
Age (years)	35.68 (4.84)	24.42 (2.68)	−13.59 ***
Years of education	11.34 (1.76)	13.10 (1.13)	5.32 ***
Duration of drug use (years)	7.06 (4.39)	/	/
Monthly drug use before rehabilitation (g)	8.87 (3.10)	/	/

*Note*: ^a^ represents the mean (standard deviation), *** *p* < 0.001.

**Table 2:** Descriptive Statistical Results of Task 1: Intra-dimension task switching.

	Methamphetamine-Dependent Group ^a^	Control Group ^b^	*F*	*p*
RT (Switch)	1.991 (0.614)	1.102 (0.198)	20.75	<0.001
RT (Repeat)	1.372 (0.423)	0.807 (0.185)	23.49	<0.001
Accuracy (Switch)	0.764 (0.224)	0.942 (0.040)	7.25	0.009
Accuracy (Repeat)	0.799 (0.206)	0.971 (0.031)	11.53	0.001
RT Switch Cost	0.619 (0.400)	0.295 (0.124)	2.92	0.09
Accuracy Switch Cost	0.035 (0.086)	0.029 (0.030)	1.15	0.29

*Note*: ^a^ Mean (*SD*) for the methamphetamine-dependent group; ^b^ Mean (SD) for the control group.

**Table 3:** Descriptive Statistical Results of Task 2: Extra-dimension task switching.

	Methamphetamine-Dependent Group ^a^	Control Group ^b^	*F*	*p*
RT (Switch)	1.593 (0.369)	1.210 (0.268)	13.13	<0.001
RT (Repeat)	1.250 (0.306)	0.841 (0.180)	26.97	<0.001
Accuracy (Switch)	0.877 (0.127)	0.958 (0.035)	3.37	0.07
Accuracy (Repeat)	0.901 (0.124)	0.968 (0.030)	1.60	0.21
RT Switch Cost	0.343 (0.182)	0.369 (0.155)	0.63	0.43
Accuracy Switch Cost	0.024 (0.030)	0.009 (0.037)	3.00	0.08

*Note*: ^a^ Mean (SD) for the methamphetamine-dependent group; ^b^ Mean (SD) for the control group.

**Table 4:** Descriptive Statistical Results of Task 3: Wisconsin Card Sorting Test (WCST).

	Methamphetamine-Dependent Group ^a^	Control Group ^b^	*F*	*p*
Failures to Maintain Set	0.366 (0.662)	0.488 (0.597)	2.43	0.12
Trials to Complete First Category	6.310 (0.897)	6.122 (0.900)	0.01	0.94
**Perseverative Errors**	2.738 (1.499)	1.659 (1.237)	2.96	0.08
Non-Perseverative Errors	10.262 (3.513)	8.100 (3.087)	8.65	0.004 **

*Note*: ^a^ Mean (*SD*) for the methamphetamine-dependent group; ^b^ Mean (*SD*) for the control group. ** *p* < 0.01.

**Table 5:** Summary of Research Results.

Assessment Measures	Methamphetamine-Dependent Participants in Reference to Controls
Intra-Dimensional Task Switching	Marginally greater RT switch costs
Extra-Dimensional Task Switching	Marginally greater accuracy switch costs
Wisconsin Card Sorting Test	Marginally more perseverative and significantly more non-perseverative errors
Cognitive Flexibility Inventory	Significantly lower self-reported scores

## Data Availability

The data presented in this study are available upon request from the corresponding author. The data are not publicly available due to participant confidentiality and institutional ethical guidelines.
